# How Frequently Do We Touch Facial T-Zone: A Systematic Review

**DOI:** 10.5334/aogh.2956

**Published:** 2020-07-06

**Authors:** Juma Rahman, Jubayer Mumin, Bapon Fakhruddin

**Affiliations:** 1 School of Population Health, The University of Auckland, Auckland, NZ; 2 International Organization for Migration (IOM), United Nations Migration Agency, BD; 3 The University of Auckland, Auckland, NZ

## Abstract

**Background:**

Researchers across the world are emphasising the importance of hand-washing and limited touching of face to curb the spread of COVID-19. However, access to safe water and hygiene is inadequate in many places around the globe; hence T-zone touching restriction is considered more worthwhile compared to other prevention strategies.

**Aim:**

A systematic review was carried out to appraise the frequency of T-zone (eyes, nose, mouth, chin) touching in humans to comprehend the challenge of its restriction, and thus support public health professionals to produce evidence synthesis guidance for public.

For this systemic review, data were collected by keyword searching, and several online databases were searched. The PRISMA checklist, PECO protocol and STROBE guideline were followed in this review, and pooled data were analysed in R version 4.

**Result:**

Total of 10 single arms observational studies were included. The pooled average (SD) facial self-touch per hour was 50.06 (±47) times, and a specific touch of T-zone was 68.7 (±27). T-zone self-touch within the total facial self-touch was found higher R = 0.680, with 95% CI 0.14, 0.91, P = 0.02 and X^2^ = 167.63, P < 0.0001.

**Conclusion:**

The review found that face-touch is a type of consistent regulatory movements. Control of T-zone touch requires extensive behaviour intervention and community awareness.

## 1 Introduction

Face touching^1^ is an expression of liberating the hands of bipedalism and one of the manipulative gestures of human behaviour [2]. Rubbing eyes, scratching nose, curling fingers against mouth or chin, chin resting on a hand (‘Rodin’s thinker’) are all distinctive taxonomies of face-touching in primates [3]. However, in recent pandemic shocks, facial self-touching quavers public health experts due to the chance of self-inoculation.

Self-inoculation^2^ identified as one of the main routes of entry of respiratory viruses [4,5]. The novel coronavirus of COVID-19 enters through the mucous membranes of eyes, nose and mouth (i.e. facial T-zone), mostly by self-inoculation [6]. Since the evolution of H1N1 flu, several pieces of research recommended that fewer T-zone touching results in a lower chance of respiratory tract infections [4,7,8]. This is because our hands remain clean until we touch the next surface, which is a fugitive state [9].

Viruses are obligatory intracellular parasites that require host cells to continue their life cycle. There are two primary pathways to enter into the host cells: one is delivering genomes to the cytosol by fusion of their envelope with the host cell, and another is the endocytic mechanism [4,10]. It is believed that coronavirus (CoV) enters cells by endocytosis pathway. It enters cells of mucous membranes via angiotensin-converting enzyme 2 (ACE2) receptors, a functional receptor of CoV [4] and replicates in mucous membranes of the upper respiratory tract before entering the lungs. Hence not touching the T-zone is predicted to be one of the life-saving behaviours without any cost associated. Researchers have studied self-touch in humans for several decades, mostly to explore brain functionality and to learn psychology. After that, it has been researched due to the emergence of CoV (SARS, H1N1, MARS, COVID-19) in recent years. A study [11] examining EEG (electroencephalography) changes caused by spontaneous facial self-touch elaborated that emotional and cognitive processes were highly relevant for the behaviours of self-face touching. Modern studies interpreted hand function as reflections of cerebral activity [3].

Given that, we have hypothesised that spontaneous self-face touch is induced with little or no conscious awareness and that it is one of the challenging behaviours to control. We conducted a systematic review to comprehend the interactive nature of face touching in humans, and by doing so, we strained to emphasise on behaviour alteration domains.

## 2 Methods

### 2.1 Search strategy

Several online databases were searched, including Ovid Medline (PubMed, Embase, Scopus), Science Direct, Auckland University Library (online), EBSCOhost, Google Scholar, the Web of Science and Cochrane Central Register of Controlled Trial. The keywords and search modifiers used were: “face touch”, “face” OR “face” OR “self” AND “touching” OR “epidemiology” OR “frequency” OR “epidemiology” AND “COVID”, “respiratory illness” “CoV” “SARS”. In addition, A hand search (i.e., manual process of screening pre-defined and pre-selected peer-reviewed journals and other publications) of the references of included studies was also conducted. Several international gold-standard methodologies were implemented in this review, including PECO (participants, Exposure, comparison, and outcomes) [12], SYRINA (Systematic Review and Integrated Assessment) [13], and PRISMA (Preferred Reporting Items for Systematic Reviews and Meta-Analyses) checklist [14].

### 2.2 Eligibility criteria

The following inclusion criteria were considered: 1) participants: humans, both sexes, and all age groups were included; 2) exposure: touching face including T-zone; 3) outcome: frequency of touching; 4) publication date: there was no restriction on publication date. The closing updated search was carried out in May 2020; 5) language: no language restriction was applied to avoid publication bias; 6) study design: all type of study designs were accepted. All kinds of research papers were included: full publications, letters, conference papers, and theses. The exclusion criteria included: 1) study design: animal studies only; 2) participants: animals only; 3) exposure: touching of other parts of the body; 4) outcome: papers described participants’ knowledge only, and did not include any findings.

### 2.3 Data extraction

Reviewer (JR) perused the titles and abstracts of citations identified in the search, and full manuscripts of potentially eligible articles were retrieved for review after removing the duplicates. EndNote X9 was used to remove the duplicates. Reviewer (JM) extracted the data following the STROBE (Strengthening the Reporting of Observational Studies in Epidemiology) [15] statement guideline for observational studies. Reviewers (JR, JM, BF) resolved any disputes by discussion and recurring scrutinising. The data items pulled out from the eligible paper included: title, author, year of publication, location of study, study settings, method of measurement, statistical method, sample size and characteristics, confounders/bias adjustment, and main results.

### 2.4 Data mining and statistical analysis

Raw data were processed into useful standard meta-data for analysis. All outcomes were transformed to “face touch per hour” in the review. Pooled data from the included studies were analysed using several statistical methods in R version 4. A Gaussian distribution was applied to understand the proportion of the scores which lie over a certain interval with high confidence based on other research conducted. Principal Component Analysis (PCA) was used to emphasise variation and boxplot was constructed to visualise the data. Hypothesis testing was done by Chi-square analyses and one-way ANOVA test. Pearson’s correlation coefficient was conducted to find the degree of correlation between face touch and T-zone touch.

### 2.5 Quality appraisal

Observational studies were assessed using a modification of the Newcastle-Ottawa Scale for use with cross-sectional and cohort studies [11]. Cross-sectional studies had a potential maximum of five stars.

## 3 Results

### 3.1 Study selection

**Figure 1:**
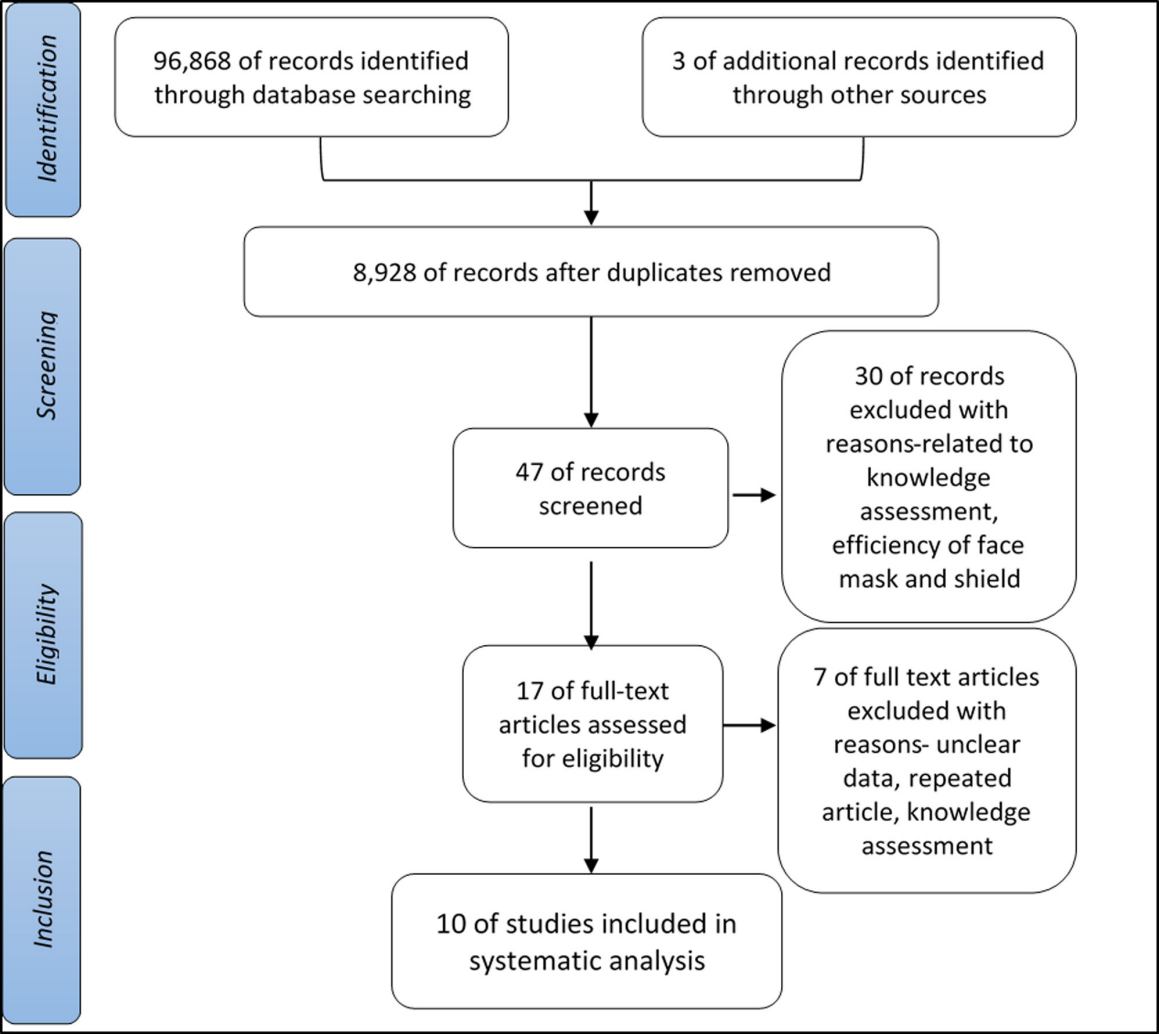
Diagram of the PRISMA flow chart showing a selection of observational studies for review.

Figure 1 shows the details of the study selection. Through database search and other sources (such as magazines, newsletters, and tabloids), a total of 96,871 studies were retrieved. However, after removing duplicates, 8,928 reviews were included for screening. Title and abstract were screened applying the inclusion and exclusion criteria, assigning 47 for full-text review. For non-English studies, Google Translate was used for translating one French journal article. Seventeen publications were carefully reviewed, and seven were excluded due to unclear data and repetition of the same study. Finally, 10 observational studies published between 1973 to 2019 were included in the subsequent review.

### 3.2 Study characteristics

Included studies were single-armed observational pieces of research, and participants were university students or office employees, excluding one study [16] that took place at a petting zoo and surveyed the frequency of face touching in public visitors. Six out of 10 studies were from the USA [1,5,7,16,17,18], one from the UK [2], one from Australia [19], one from Japan [20], and the later one [21] took place in Japan (Osaka University) and UK (Cardiff University). The participants of six studies [1,2,17,19,20,21] were university students, and the researchers observed them during lecture time or assigned work; two studies involved health professionals [7] and medical students [19] while dealing patients; one study included public visitors [16], others involved researchers [18] and employees [5]. The number of participants in most studies was less than 50, but one [16] included 574 participants.

### 3.3 Summary of study results

Overall, the quality assessment of the publications based on the Newcastle-Ottawa Scale of these studies was rated as satisfactory, good or very good. Table 1 shows the result of the quality assessment of included studies. The included observational studies reporting results are summarised in Table 2. The aim of most of the studies was the observation of the frequency of face touch. One study used this outcome to investigate the cross-cultural cerebral function [21], one study to examine the accuracy of the self-report [1]. At the same time, one study compared the behaviour with primates [2], and some to evaluate the application of the hand hygiene concept in reality [7,19]. Participants of all studies were blinded except in four studies [1,17,18,20]; one of them [1] compared the accuracy and reactivity of self-monitoring, so the participants knew the purpose of the study. However, we have extracted the baseline data for this study [1] where two researchers observed the frequency of their face touch and they were blinded. In the other three studies [17,18,20], participants were informed that they were surveyed, but they were not particularly well-versed about the face self-touching inspection. Three studies [17,19,20] used videotape recording, while two [2,21] used PET computing facilities and magnetic tape data recorder. Another study from Japan took place in a simulated train cabin and was video monitored [20].

**Table 1:** Quality assessment of the included studies based on the Newcastle Ottawa scale.

	Hendley, 1973	Nelson, 1982	Diamond, 1984	Hatta, 1984	Nicas, 2008	Erdozain, 2011	Elder, 2014	Johnston, 2014	Kwok, 2015	Morito, 2019

**Selection**										
1) Representativeness of the exposed group.	c (0)	d (0)	b (+1)	b (+1)	c (0)	a (+1)	b (+1)	c (0)	b (+1)	b (+1)
a) Truly representative of the average person in community*										
b) Somewhat representative of the average person in community*										
c) Selected group of users										
d) No description of the derivation of the group										
2) Selection of the non-exposed group.	c (0)	c (0)	c (0)	c (0)	c (0)	c (0)	c (0)	c (0)	c (0)	c (0)
a) Drawn from the same community as the exposed group*										
b) Drawn from a different source										
c) No description of the derivation of the non-exposed group										
3) Ascertainment of exposure.	b (+1)	b (+1)	b (+1)	b (+1)	b (+1)	b (+1)	b (+1)	b (+1)	b (+1)	b (+1)
a) Secured record (e.g. lab)*										
b) Structured interview or questionnaire*										
c) Written self-reports										
d) No description										
**Confounder**										
1) Comparability of groups on the basis of the design or analysis.	b (+1)	b (+1)	b (+1)	b (+1)	b (+1)	b (+1) a (+1)	b (+1)	b (+1) a (+1)	b (+1)	b (+1) a (+1)
a) Study controls for age and sex*										
b) Study controls for any additional factor*										
**Outcome**										
1) Assessment of outcome.	a (+1)	a (+1)	a (+1)	a (+1)	a (+1)	a (+1)	a (+1)	a (+1)	a (+1)	a (+1)
a) Independent blind assessment*										
b) Record linkage*										
c) Self reports										
d) No description										
**Overall Score (out of 5)**	3 Satisfactory	3 Satisfactory	4 Good	4 Good	3 Satisfactory	5 very good	4 Good	4 Good	4 Good	5 very good

**Table 2:** Summary of the included studies.

Study ID and country	Study sample	Time of observation	Methods of measurement	Outcome	Part of the face touched

Hendley (5), USA	89, employees of an Insurance company and their families.	60	Observation	Total 62 times touched per hour. 1/3 of total touch was picking noses, 1/2.7 rubbing eyes.	Nose and eyes. Nose was touched more than eyes.
Nelson (1), USA	16, students	4hr, 9 hr	Observation	mean frequency of touch 13.09 per 5-min interval.	Any part including neck and earrings with pens and water container.
Dimond (2), UK	18, students	30	PET computer conducted	mean 13.33 times per 20 minutes.	Mouth (18%), chin (57%), and nose were touched.
Hatta (21), Japan and UK	36, students	30	PET computer and magnetic tape data recorder	They were observed in 3 different strata- with no task, listening to music and lecture. For Japanese 4.5, 2.6 and 3 times and British 13.6, 8.8 and 9 times respectively.	Japanese/British Mouth = 17.5/18.3 Chin = 17.5/58.0 Cheek = 7.0/3.4 Nose = 24.6/10.6 Scalp = 8.8/4.8 Ear = 5.3/0.6 Forehead = 5.3/1.4 Eye = 14.0/2.8
Nicas (17), USA	10, students	180	Videotape recording	15.7 per hour.	Eyes = 7.4, Lips/mouth = 24, Nose = 16 times.
Erdozain(16), USA	574, public visitors to animal petting zoos.	30	Observation	Children 77%, adults 69% touched face in total self-touch.	Not specified.
Elder (7), USA	79, health personnel	120	Observation	19 times in two hours.	The mouth was touched twice as often as other parts (nose/eyes).
Johnston (18), USA	93, employees and students from a laboratory.	337 (average)	Observation		Nose 44.9%, Mouth 4%, Eye 1.7%, Forehead 36.9% and Cheek/Chin 12.5%.
Kwok (19), Australia	26, medical students	120	Videotape recording	23 times per hour.	44% = T-zone (36% = mouth, 31% = nose, 27% = eyes, and 6% = combination of these regions) and 56% = non-mucosal areas.
Morita (20), Japan	40 students	30	Video monitoring in a simulated cabin	17.8 times per hour.	T-zone = 42.2% and 57.8% = non-mucosal surface.

Face touching pattern showed differences in sex, age, hand domination and culture. Europeans were found touching face more frequently than Asians; they touched chin and mouth mostly while their counter group touched nose and eyes [21]. There were no differences in frequency in sex and hand domination; male and female frequently touched the face [16,20,21]; also, there was no difference in left-handed and right-handed participants [2]. However, sex differences were observed only in the presence of wearing cosmetics [20]. Participants who had less hand hygiene awareness were often found touching their face [7,18,20]. Health professionals (doctors, nurses, laboratory technicians) were more aware of self-touch than other staffs [7,18]. More remarkably, one study conveyed the differences in point of touch being dependant on public visibility. Participants touched or picked nose more in the amphitheatre arrangement than in the conference sitting plan [5]. The spontaneous self-face touch was compared to this behaviour in apes in one study, and the pattern of face touching was found comparable to gorillas, orangutans and chimpanzees [2]. The use of left hand to touch face was frequent [2,16], which supports the conjecture of emotional dominance in the right cerebral hemisphere and cortical functioning [3,11]. Face touch frequency also differs with the nature of the task. No task, listening to music, smartphone use, emotions, memory task were found related to spontaneous self-touch [5,17,20,21].

### 3.4 Results of pooled data

**Figure 2:**
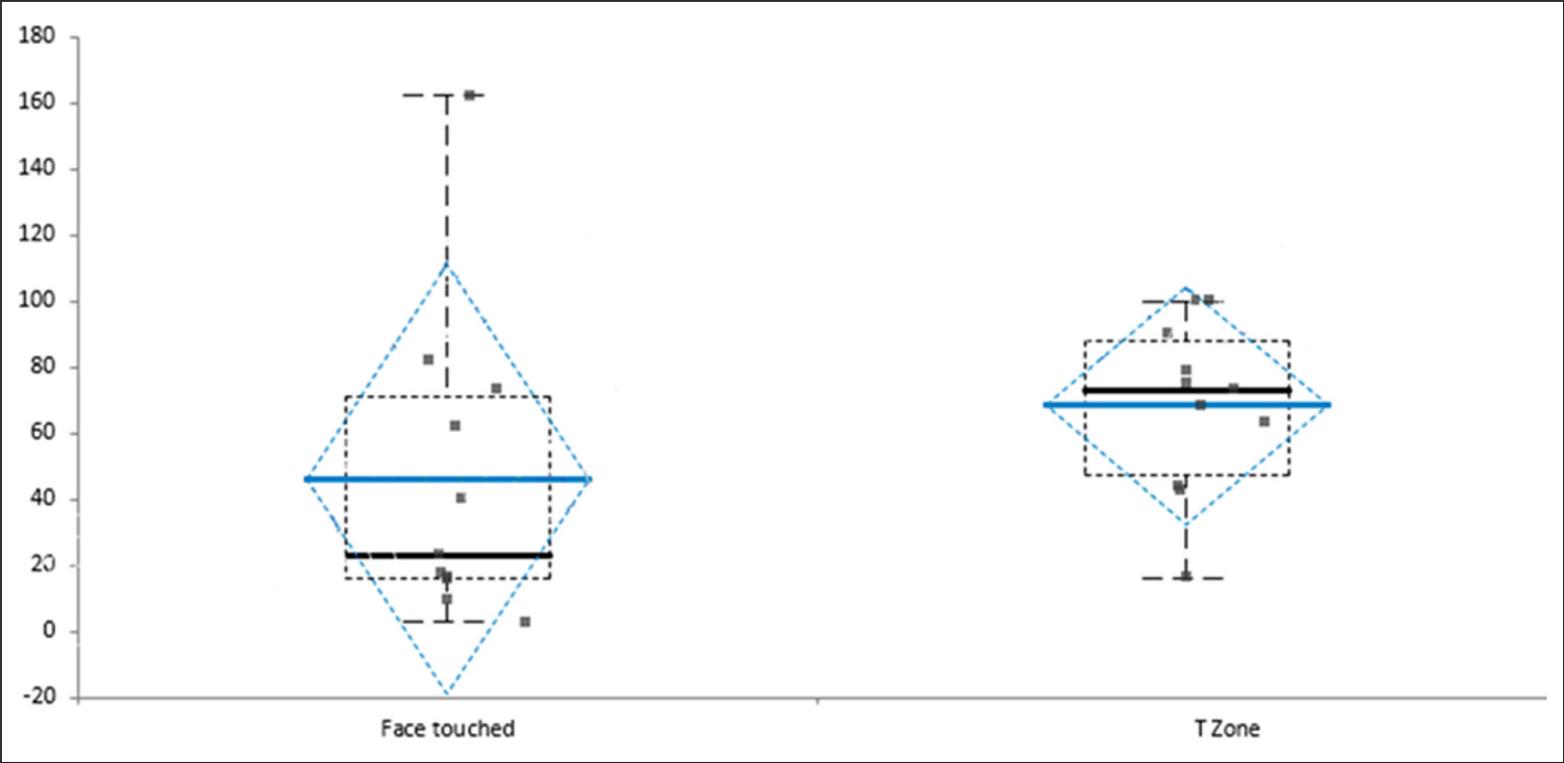
The distribution of the standard deviation of T-zone touch.

Average touch of self-face per hour was 50.07 times (SD = 47), and T-zone (eyes, nose, mouth and chin) was 68.7 (27). Detailed results were presented in Table 3, and the distribution of the standard deviation of T-zone touch stressed in Figure 2. There was a lack of symmetry in the data distribution. The skewness factor was –0.05, which indicates that a distribution of the database did not follow any normal distribution. Kurtosis value for the T-zone was –0.89, indicating a lack of outliers in the database as the extreme values are less than those of normal distribution.

**Table 3:** Mean (SD) of frequency of face and T-zone touch.

Variables	Mean	SD	Minimum	Median	Maximum

Face touched per hour	50.06	47.2	9.5	31.5	162
T-zone touched per hour	68.70	27.2	16	74	100

The correlation of T-zone touched in the total face touch was compared with various statistical methods and shown in the Table 4. Correlation coefficient R = 0.680 with 95% CI 0.14, 0.91, P = 0.02.

**Table 4:** Correlation coefficient of face touched within T-zone per hour.

	T-zone	Correlation coefficient

**Face touched**	0.669	Pearson’s R
	0.620	Spearman’s rs
	0.494	Kendall’s tau

The one-way analysis of variance (ANOVA) for the face touched in T-zone is presented in Table 6. The comparisons shown borderline significance value (P = 0.0643). The Chi-square test for the given probabilities for mouth, nose, eyes separately, and T-zone touch are elaborated in Table 5. Assuming an alpha of 0.05, we reject the null hypothesis.

**Table 5:** Chi-square tests showing the frequency of face touch in humans.

Parts of face	Chi-square value	df	P-value

Eyes	163.11	10	<0.0001
Nose	160.67	10	<0.0001
Mouth	164.71	10	<0.0001
T-zone	167.63	10	<0.0001

**Table 6:** The ANOVA test for the T-zone touch (N = 11).

Source	Sum of square (SS)	Degree of freedom (df)	Mean squares (MS)	F	p-value

Measures	2.768872E+03	1	2.768872E+03	4.32	0.0643*
Subjects	2.238206E+04	10	2.238206E+03		
Error or residual	6.408358E+03	10	6.408358E+02		
Total	3.155929E+04	21	1.502823E+03		

Where: H0: μ1 = μ2 = μ… The mean of the populations are all equal. H1: μi ≠ μj for at least one i,j. The mean of the populations are not all equal. * Do not reject the null hypothesis at the 0.1% significance level.

**Figure 3:**
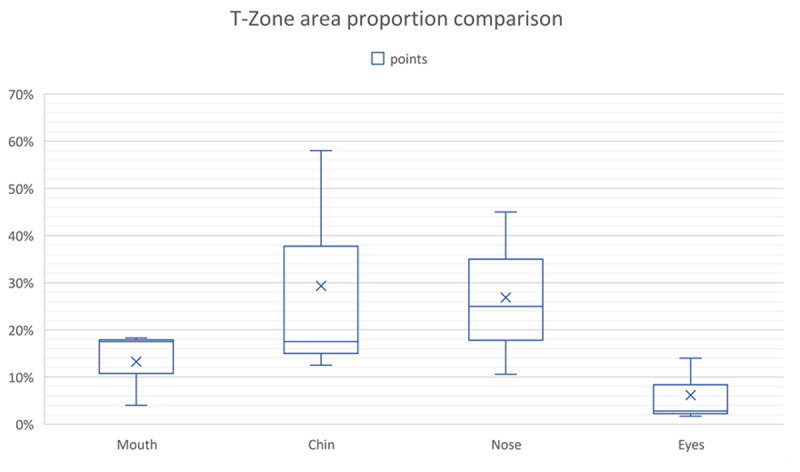
T-zone area proportion comparison based on mean and standard deviation (SD).

**Figure 4:**
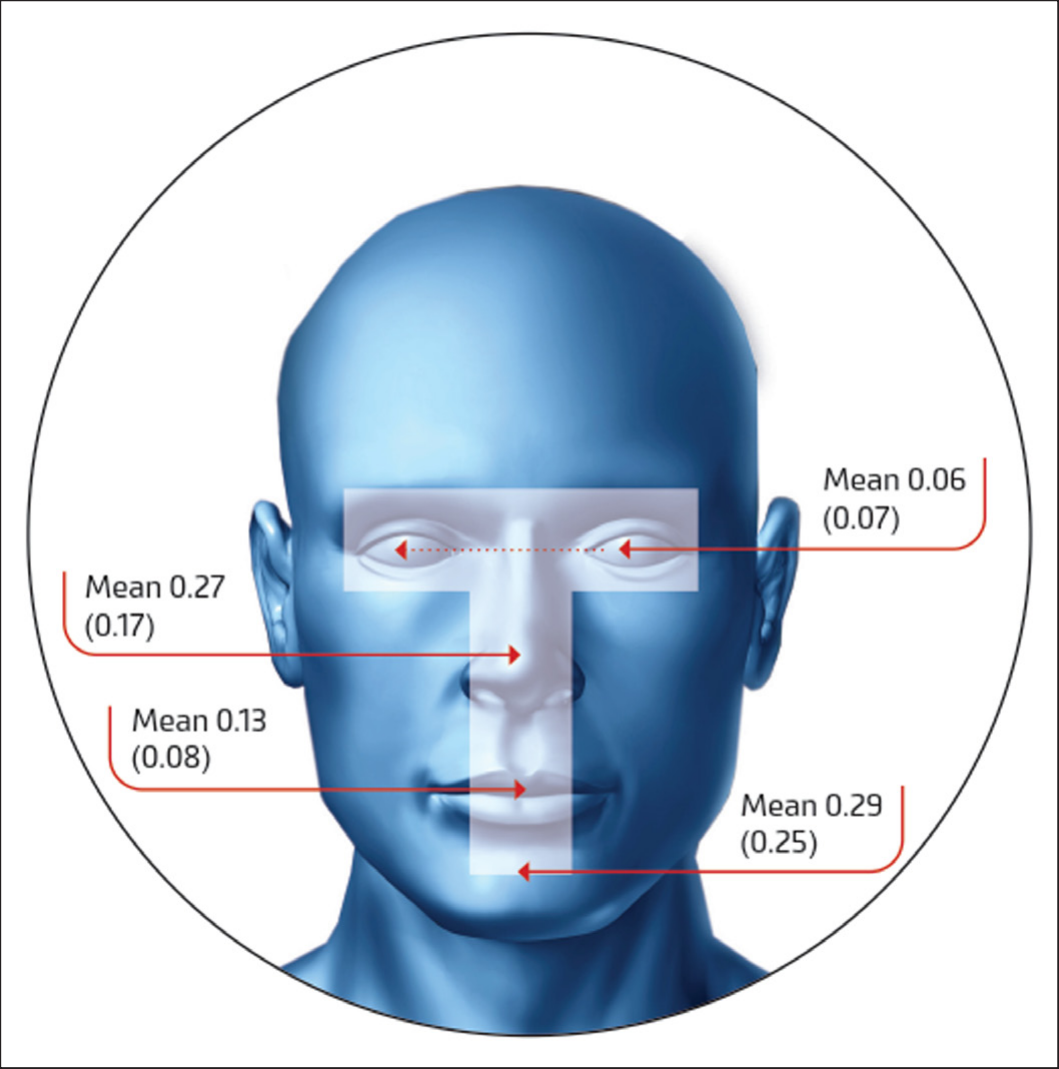
Mean (SD) of the T-zone touch (pooled data).

The mean value for the components of T-zone is summarised in Figure 3. It was found, based on the sample study, that chin (29%) and nose (27%) were frequently touched compared to other parts of the face. The overall mean and SD distributions are shown in the Figure 4.

## 4 Discussion

This review has identified a small body of evidence, primarily from observational studies, which helped us to produce a conjecture that the prevention of self-inoculation of COVID-19 requires extensive behaviour control approach to avoid pandemic related economic and health consequences. Overall, this review has recommended that face touch is elicited in every human being without any stimulation and is a type of consistent regulatory movements like posture changing. This is not to suggest that self-touching should be recognised; self-touch exhibits not only a person’s anxiety or uneasiness but mostly some comprehensive state of emotional and working memory homeostasis [11]. Hand hygiene (i.e. hand wash), social distancing and avoiding T-zone touch are suggested by the clinical researchers as fundamental instrumental acts to defend from COVID-19. However, in cases of running water scarcity, hand-washing strategy to reduce transmission of infection will be ineffective [22,23]. Similarly, social distancing makes people slow down and reconsider several issues that they take for granted, but could increase anxiety, stress and other consequences [24,25]. Relatively, awareness of T-zone touch could significantly reduce the infection rate.

To increase compliance with public health speak may need to introduce where the message could include both positive descriptive norms (information on desirable typical behaviour) and prescriptive injunctive norms (social approval for such action). This can be achieved by targeting behaviour alteration domains: environment, habits and motivations. Scientists recommend several ways to implement these practices: counter habit (i.e., training to redirect the impulses), behavioural and physical barriers (e.g., hand clasped sitting posture, wearing makeup/masks/face shields/hand gloves), and mindfulness (e.g., wearing hand gloves/perfumes in hands to remind as the hand is nearer to the face) [9].

In conclusion, the results of this review suggest that without reinforcing specific “behaviour control approach” T-zone touch restriction, which is the only beneficial approach compared to other mitigations, would be a highly challenging intrigue in public health.

## Footnotes

“*Face-touching* was defined as touching any part of one’s face, head, hair or neck (or extension of this area, such as glasses or earrings) above an imaginary plane resting on the shoulders, with one’s hand (or extensions of the hand, such as a pen or drink container)” Nelson RO, Boykin RA, Hayes SCJBr, therapy. Long-term effects of self-monitoring on reactivity and on accuracy. 1982; 20(4): 357–63.

When a person’s contaminated hand/s transmit germs from one part of the body to another, it is termed as *self-inoculation*.

## Abbreviations

CoV = Coronavirus

COVID-19 = Coronavirus Disease 2019

SARS = Severe Acute Respiratory Syndrome

MERS = Middle Eastern Respiratory Syndrome

NOS = Newcastle-Ottawa Scale

STROBE = STrengthening the Reporting of OBservational studies in Epidemiology
